# XAI-Based Assessment of the AMURA Model for Detecting Amyloid-β and Tau Microstructural Signatures in Alzheimer’s Disease

**DOI:** 10.1109/JTEHM.2024.3430035

**Published:** 2024-07-17

**Authors:** Lorenza Brusini, Federica Cruciani, Gabriele Dall’Aglio, Tommaso Zajac, Ilaria Boscolo Galazzo, Mauro Zucchelli, Gloria Menegaz

**Affiliations:** Department of Engineering for Innovation MedicineUniversity of Verona 19051 Verona 37134 Italy; Department of Computer ScienceUniversity of Verona 19051 Verona 37134 Italy; Department of Research and Development Advanced ApplicationsOlea Medical La Ciotat 13600 France

**Keywords:** Amiloyd-beta, tau, AMURA, tract-based spatial statistics, eXplainable Artificial Intelligence

## Abstract

Brain microstructural changes already occur in the earliest phases of Alzheimer’s disease (AD) as evidenced in diffusion magnetic resonance imaging (dMRI) literature. This study investigates the potential of the novel dMRI Apparent Measures Using Reduced Acquisitions (AMURA) as imaging markers for capturing such tissue modifications.Tract-based spatial statistics (TBSS) and support vector machines (SVMs) based on different measures were exploited to distinguish between amyloid-beta/tau negative (A $\beta $-/tau-) and A $\beta $+/tau+ or A $\beta $+/tau- subjects. Moreover, eXplainable Artificial Intelligence (XAI) was used to highlight the most influential features in the SVMs classifications and to validate the results by seeing the explanations’ recurrence across different methods.TBSS analysis revealed significant differences between A $\beta $-/tau- and other groups in line with the literature. The best SVM classification performance reached an accuracy of 0.73 by using advanced measures compared to more standard ones. Moreover, the explainability analysis suggested the results’ stability and the central role of the cingulum to show early sign of AD.By relying on SVM classification and XAI interpretation of the outcomes, AMURA indices can be considered viable markers for amyloid and tau pathology. Clinical impact: This pre-clinical research revealed AMURA indices as viable imaging markers for timely AD diagnosis by acquiring clinically feasible dMR images, with advantages compared to more invasive methods employed nowadays.

## I. Introduction

Amyloid-beta (A $\beta $) accumulation and neurofibrillary tangles due to phosphorylated tau protein define the Alzheimer’s disease (AD) molecular pathology [1]. Recent studies showed that both can occur from a pre-clinical and asymptomatic condition to the appearance of symptoms such as mild cognitive impairment (MCI) and diagnosed dementia [2]. For this reason, markers reflecting these two targets’ variations in the earliest disease phase are currently researched to develop potential therapies able to slow or stop its progression. At present, A $\beta $ and tau can be revealed by positron emission tomography and cerebrospinal fluid via lumbar puncture [3]. However, more advantageous methods are required since these techniques are used at the cost of radioactive tracers, high spending and invasiveness [3]. Recent pathology studies employing diffusion magnetic resonance imaging (dMRI) reported brain microstructural abnormalities in the earliest phases of AD in both gray (GM) [4] and white matter (WM) [3], [5], [6], [7]. In particular, such abnormalities can be detected before the appearance of brain atrophy typically evidenced by classical T1-weighted (T1w) MRI [4], [5].

The diffusion tensor imaging (DTI) [8] is the most popular dMRI model used in clinics to quantitatively measure microstructural brain tissue properties. It was recently exploited by Chen et al. [3] to characterize WM differences among A $\beta $-negative/tau-negative (A $\beta -$/tau−), A $\beta $-positive/tau-negative (A $\beta +$/tau−), and A $\beta $-positive/tau-positive (A $\beta +$/tau+) cognitively normal controls (CN), as well as A $\beta +$/tau+ MCI and AD subjects. They found widespread WM alterations in the whole AD continuum, but such a finding occurred especially early and correlated with tau pathology in the *hippocampal cingulum* revealing the potential of dMRI in the early AD detection challenge. The DTI popularity is mainly due to its simplicity and feasibility with data acquired in clinical conditions (*i.e.*, low number of diffusion gradients in addition to their poor strength and timing). Nevertheless, it assumes a Gaussian trend of the dMRI signal with the intrinsic limitation of failing the reconstruction of complex WM architectural configurations where diffusion is anisotropic (*e.g.*, fibers’ crossings, kissings, etc.). Recent overcomes of this limitation are represented by multi-shell dMRI and new modelling techniques like the Neurite Orientation Dispersion and Density Imaging (NODDI) [9], though not standard for clinical purposes. NODDI is a compartmental model which assumes that the brain tissue is divided in isotropic, intracellular diffusion, and extracellular microstructural compartments. Vogt et al. [6] showed that the NODDI-derived neurite density index was sensitive to GM modifications in A $\beta +$/tau+ CN before the onset of brain atrophy and cognitive impairment. Spotorno et al. [4] too demonstrated the higher potential of GM microstructural alterations in revealing the astrocytic response to A $\beta $ aggregation compared to macrostructural measurements as those derived from T1w-MRI. In particular, they relied on a multi-shell acquisition as in [6], but they employed the Mean Apparent Propagator (MAP)MRI model [10] which, differently from NODDI, does not assume any tissue composition. Indeed, avoiding assumptions on tissue composition is preferable especially in disease state because the underlying biophysical theoretical assumptions at the bases of the compartmental models probably do not hold in such conditions. In line with this consideration, Moody et al. [7] used MAPMRI to detect AD-related early neurodegenerative changes in WM, finding more spatially diffuse associations with A $\beta $ and tau cerebrospinal fluid markers compared to DTI and NODDI.

In the present study, we further investigated the potential of dMRI in highlighting WM microstructural alterations in the earliest phases of AD with the twofold goal of exploiting typical clinical acquisition protocols while enabling finer microstructural characterization. To this end, we relied on a recently proposed method called Apparent Measures Using Reduced Acquisitions (AMURA) [11], allowing to exploit single-shell acquisition protocols while maintaining the descriptive power of MAPMRI indices under certain conditions. AMURA applied to high *b*-value acquisitions provides microstructural indices with similar sensitivity compared to MAPMRI-derived ones [11], hence allowing a highly specific microstructural characterization at a finer granularity compared to DTI while bringing DTI-like advantages such as clinical feasibility, *i.e.* a reduced number of samples and low computational complexity. In this preliminary study, AMURA capability in the characterization of different A $\beta $ and tau status in the AD continuum was compared with the classical DTI in exquisitely clinical acquisitions (*i.e.* low *b*-value) allowing to test its suitability also in such a scenario. Of course, the complete characterization of the method would require to contrast it with the MAPMRI outcomes when relaxing the constraint of single-shell acquisitions. However, this is out of the scope of this contribution which focuses on classical single-shell acquisitions for which DTI is the *de-facto* benchmark. Though the applied method is not new, in our opinion its application to a hard and open problem at the state of the art also holding high translational potential deserves an in-depth analysis, and the *post-hoc* assessment of the outcomes would mark a step in the direction of the neurophysiological plausibility of the results, and thus on the relevance and usefulness of the method in the translational perspective highlighting its ability to capturing actual tissue alterations without being invasive.

In the context of *post-hoc* assessments, the application of eXplainable Artificial Intelligence (XAI) methods is gaining increasing importance due to the outbreak of AI applied to the biomedical field in the last years. Indeed, XAI has the potential of offering a key for the interpretation and strengthening of the AI-derived results themselves by bringing to light some aspects of the internal thinking of complex models, deep networks on the top of the list, or providing ordered lists of input features leading the algorithm’s outcomes. Many examples can be found in the literature [12], [13], though a real awareness of the intrinsic limitations and related risks of such methods is rarely acknowledged and faced. Otherwise stated, the validation of the XAI method is most often overlooked, that is a serious risk especially in the biomedical field. In this work, in addition to employ AI and XAI to assess the early AD classification when based on different microstructural properties of the tissue, we faced the validation issue by comparing two different interpretability methods and through a more simple framework that is the relying on the prior knowledge derived from the literature. The last is a qualitative validation of the outcomes limited to the neurophysiological plausibility. Other validation strategies like *post-hoc* association studies as well as the analysis of other attributes of XAI methods are left for future investigation. In particular, in this work we rely on SHAP [14] and LIME [15] methods, that are among the most widespread for their conceptual simplicity and understandability, to derive an ordered list of features, *i.e.* brain WM tracts, allowing to discriminate across the A $\beta $ and tau spectra. To the best of our knowledge, only one work which specifically investigated the pre-clinical AD attempted to give an interpretation of the results by exploiting the XAI. In the mentioned study, Hwang et al. [16] classified A $\beta $+ and A $\beta $- CN with a deep generative model relying on T1w scans and many other features like demographics and cognitive scores. They subsequently used the integrated gradients XAI method to explain their outcomes. Hence, the main novelty represented by our current investigation consists of the information used at the basis of the classification. Indeed, the dMRI targets the microstructural properties of the tissue instead of the macrostructural ones as the T1w-MRI does. In this respect, this work constitutes a step forward in the comprehension of the mechanisms at the basis of AD, also thanks to the translational power of the XAI that provides reliable explanations of easy interpretation for the clinicians.

## II. Methodology

### A. Microstructural Brain Description Through AMURA

AMURA is an innovative method for studying cerebral microstructure [11]. The innovation is represented by the capability of computing ensemble average propagator (EAP)-based indices such as the return to origin/axis/plane probability (RTOP/RTAP/RTPP) with lower computational complexity and number of diffusion gradients compared to current state-of-the-art approaches like MAPMRI [10]. These three indices represent the probability that protons do not move during the dMRI acquisition, thus reflecting barriers’ restriction and consequently cell bodies’ size measures. In particular, RTAP and RTPP can be considered as the RTOP projections on the perpendicular plan and parallel direction to the maximum diffusion. The simpler computation with AMURA is possible because it treats the diffusion anisotropy as independent on the *b*-value (*i.e.*, the factor indicating the acquisition diffusion gradients’ strength and timing). Given $b=q^{2}\tau $, the normalised signal $E(\mathbf {q})$ in the **q**-space can be formalized as:

\begin{equation*} E(\mathbf {q}) = E(q_{0}, \theta, \phi) = \exp \bigl (-4\pi ^{2}\tau q_{0}^{2}D(\theta,\phi)\bigr), \tag {1}\end{equation*}

where $\mathbf {q}=q\mathbf {u}$ ( $\mathbf {u}\in \mathcal {S}$ is a unit direction in space, and $||\mathbf {u}||=1$), $q_{0}=||\mathbf {q}||$, $\theta $ and $\phi $ are the angular coordinates in the spherical system, $\tau $ is the diffusion time, and $D(\theta,\phi)$ is the apparent diffusion coefficient (ADC) on a single-shell acquisition [17]. In light of the assumptions described above, a numerical implementation of the indices based on spherical harmonics (SH) expansions has been proposed in [11]:

\begin{align*} \textrm {RTOP}_{\textrm {AMURA}} & = \frac {1}{(4\pi) ^{2} \tau ^{3/2}} C_{0,0} \{ D(\theta, \phi)^{-3/2} \}, \tag {2}\\ \textrm {RTAP}_{\textrm {AMURA}} & = \frac {1}{8\pi ^{2} \tau } \mathcal {G} \left \{{{ \frac {1}{D(\theta ')}}}\right \}(\mathbf {r_{0}}), \tag {3}\\ \textrm {RTPP}_{\textrm {AMURA}} & = \frac {1}{\sqrt {4\pi \tau }} \frac {1}{\sqrt {D_{SH}(\mathbf {r_{0}})}}. \tag {4}\end{align*}

$C_{0,0} \{ D(\theta, \phi)^{-3/2} \}$ is the $0^{th}$ order coefficient of the SH series expansion, $\mathcal {G} \left \{{{ \frac {1}{D(\theta ')}}}\right \}(\mathbf {r_{0}})$ is the Funk-Radon transform of the inverse of the diffusion signal $D(\theta ')$, *i.e.* the diffusion signal at the equator normal to $\mathbf {r_{0}}$ (the direction of maximum diffusion), parameterized by the angle $\theta '$, and $D_{SH}(\mathbf {r_{0}})$ is the SH regularized version of the ADC evaluated at $\mathbf {r_{0}}$.

### B. Dataset

Data used in the preparation of this article were obtained from the Alzheimer’s Disease Neuroimaging Initiative (ADNI) database (adni.loni.usc.edu). The ADNI was launched in 2003 as a public-private partnership, led by Principal Investigator Michael W. Weiner, MD. The primary goal of ADNI has been to test whether serial MRI, PET, other biological markers, and clinical and neuropsychological assessment can be combined to measure the progression of MCI and early AD. Images were collected from different centers with scanners from GE, Philips, and Siemens vendors. Please refer to https://adni.loni.usc.edu/ for up-to-date information. 442 subjects being CN or MCI were selected from the ADNI phase 3 (ADNI3) database. Although both basic and advanced dMRI acquisitions were performed in this phase of the study consisting of single or multiple shells acquisitions, only single-shell ones were considered for the current study [18].

For each subject, the 3D T1w-MRI volume (sagittal accelerated MPRAGE, TR/TE = shortest, TI =900 ms, FOV $= 208\times 240\times 256$ mm^3^, flip angle =9°, resolution $= 1\times 1\times 1$ mm^3^) and the single-shell dMR image (acquisition through 3T MRI scanner, TR/TE =7200/56 ms, FOV $= 232\times 232\times 160$ mm^3^, resolution $= 2\times 2\times 2$ mm^3^, ${b} =0$ and 1000 s/mm^2^, diffusion time $\tau = 38.7$ ms) were collected along with concentration values of A $\beta $ and tau protein in the cerebrospinal fluid. These concentration values were used to stratify the cohort into 3 classes. In particular, subjects were classified as A $\beta +$ if [A $\beta $-protein $] \leq 980$ pg/mL, and tau+ if [tau-protein $] \geq 24$ pg/mL (see Table 1).

**TABLE 1:** Demographic Summary of the Study Cohort

	$\text {A} {\beta } \text {-}$ tau−	$\text {A}{\beta } \text {+}$ tau−	$\text {A}{\beta } \text {+}$ tau+
N (%female)	168 (58.3%)	128 (44.5%)	146 (50%)
Age	$71.16\pm 7.16$	$74.37\pm 7.09$	$76.27\pm 8.27$

### C. Preprocessing

A minimal preprocessing including the bias-field correction and the linear registration to the 2-mm MNI space was applied to T1w images by employing the *fsl_anat* tool (FSL, version 6.0, https://fsl.fmrib.ox.ac.uk/fsl/fslwiki/) [19].

The dMRI data were preprocessed by extracting the brain and performing Eddy currents correction still using FSL [20]. Subsequently, data were denoised using the Python *dipy* library (https://dipy.org/) to apply a principal component analysis (PCA)-based denoising algorithm with automatic PCs classification grounding on the Marcenko-Pastur distribution [21] (the radius of the 3D sliding window was set equal to 2). The *b0* volumes were averaged and registered to the T1w image of the subject through the *epi_reg* routine in FSL [22], and then linearly registered to the MNI space by applying the transformation obtained through *fsl_anat*. The same linear transformations applied to the average *b0* were also applied to all the other volumes of the dMR image, and the result obtained was further non-linearly registered to the MNI space through ANTs software (http://stnava.github.io/ANTs/) [23] to correct for EPI-induced currents [24]. The dMRI gradients’ direction was rotated accordingly.

### D. Microstructural Indices Extraction

RTOP/RTAP/RTPP $_{\textrm {AMURA}}$ microstructural descriptors were derived following the numerical implementation described in Section II-A. A SH spherical order of 6 and a cost parameter for the Laplace-Beltrami regularization of $\lambda _{LB}=0.001$ were selected for the computations. In addition, relying on the *dipy* library, the diffusion tensor model [8] was used to obtain DTI-based versions of RTOP/RTAP/RTPP as follows [11]:

\begin{align*} \textrm {RTOP}_{\textrm {DTI}} & = \frac {1}{\sqrt {(4\pi \tau)^{3}}} (\lambda _{1}\cdot \lambda _{2}\cdot \lambda _{3})^{-1/2},& \tag {5}\\ \textrm {RTAP}_{\textrm {DTI}} & = \frac {1}{\sqrt {(4\pi \tau)^{2}}} (\lambda _{2}\cdot \lambda _{3})^{-1/2},& \tag {6}\\ \textrm {RTPP}_{\textrm {DTI}} & = \frac {1}{\sqrt {(4\pi \tau)}} (\lambda _{1})^{-1/2}. \tag {7}\end{align*}

Specifically, $\lambda _{i}$ is the $i^{th}$ eigenvalue of the diffusion tensor. Standard Mean Diffusivity (MD) and Fractional Anisotropy (FA) microstructural descriptors were also derived [8].

### E. Tract-Based Spatial Statistics

The tract-based spatial statistics (TBSS) pipeline from FSL was performed on the aforementioned FA images. More in detail, all images were registered to the FA image of the JHU DTI-based WM atlas [25] through a non-linear transformation, and the resulting WM skeleton was obtained using a threshold of 0.2 on the calculated average volume. The same obtained registrations were subsequently applied to the images of all microstructural indices. The pipeline ended with a two-sample unpaired *t*-test performed through the FSL tool *randomise* [26]. Both contrasts (CN > patients, and patients > CN) were investigated for each index by comparing A $\beta -$/tau− with A $\beta +$/tau−, and A $\beta -$/tau− with A $\beta +$/tau+. For each test, the number of permutations performed was 1000. Images representing the threshold-free cluster enhanced *p*-value corrected for multiple comparisons across space were obtained.

### F. Support Vector Machine-Based Classification

A $\beta -$/tau− with A $\beta +$/tau−, and A $\beta -$/tau− with A $\beta +$/tau+ were further investigated by performing their classifications through Support Vector Machines (SVMs) relying on Scikit-Learn library (https://scikit-learn.org/stable/) in Python. SVMs are notoriously able to perform classification tasks in a very versatile way (e.g., linearly or not linearly) [27], and they are commonly used for biomedical applications with good performance [28], [29]. Besides, a deep learning strategy was not affordable in this work because of the limited number of subjects available.

Considering only voxels belonging to the WM skeleton, for all subjects, the average value of each index was extracted from 48 Regions of Interest (ROIs) (*i.e.*, WM tracts) based on the JHU DTI-based WM atlas previously introduced [25]. Thus, for each of the two classification tasks, we trained and validated 8 SVMs (one per microstructural index), each one based on a different initial data matrix of dimensions $N \times 48$. *N* was the total number of subjects depending on the two classes of the classification task to handle, while 48 was the number of WM tracts.

The optimal hyperparameters were found through an exhaustive search performed with a cross-validation strategy over all possible kernels and [0.01, 0.1, 0.2, 0.3, 0.4, 0.5, 0.6, 0.7, 0.8, 0.9, 1, 10, 20] regularization parameters *C*, resulting in the linear kernel and $C = 0.4$. The final classification tasks were carried out with a stratified 10-folds cross-validation, and the performance was assessed by calculating the mean accuracy, precision, sensitivity and specificity over the folds. Due to the limited cohort numerosity, we did not retain also a test set in addition to those used for training and validating the model.

### G. Explainable Artificial Intelligence Analysis

SHAP and LIME were used for identifying the features that contributed most to the SVM outcome. To this end, we focused on the classification task generally obtaining the best performance based on validation accuracy in order to maximise the generalizability, reliability and robustness of the results [30]. Indeed, poor classification performance would be an indication of the difficulty of the model in discriminating the classes relying on the available features, casting shadows on the actual relevance of the SHAP/LIME attribution values.

Given the lack of an independent test set, for each microstructural index, the fold obtaining the best accuracy among the ten was selected in order to maximize both predictive and descriptive accuracy [30]. The former is the classifier’s accuracy, while the latter is the objective capability of the interpretability method to capture the relationships learned by the classifier itself. Both predictive and descriptive accuracies should be high to obtain a trustworthy explanation, but the former constrains the latter. For this reason such a selection was done as in [31], [32], [33], and [34]. Thus, the SHAP and LIME values indicating the relevance of each feature to the classification of every subject in the validation set were calculated.

#### 1) Shapley Additive Explanations

SHapley Additive exPlanation (SHAP) [14] is a model-agnostic and perturbation-based method for estimating the input feature importance. Basically, it is a method from coalitional game theory where a prediction is explained by assuming that each feature is a “player” in a game where the prediction is the payout. The SHAP value of a feature is calculated as the average marginal contribution of that feature across all possible coalitions. Calculating Shapley feature importance values thus becomes computationally expensive for complex models and high number of features. However, this is not the case for the problem at hand where both the number of features and data samples (subjects) is limited. SHAP has demonstrated its efficacy in the medical domain to explain clinical decision-making both from image [35], [36], [37] and non-image [38], [39], [40], [41] inputs.

In this work, the SHAP library (https://github.com/shap/ shap) in Python was used with a kernel explainer using a weighted linear regression to compute the importance of each feature. For each index and feature, the mean SHAP value over the validation set was derived.

#### 2) Local Interpretable Model-Agnostic Explanations

The Local Interpretable Model-agnostic Explanations (LIME) [15] is a model-agnostic method based on perturbation like SHAP, with the main difference of focusing on explaining individual predictions instead of providing a global interpretation based on the whole dataset. Given an individual prediction, the approach starts with the creation of a fictitious dataset produced by perturbing the corresponding input features within a proximity usually defined by an exponential kernel based on the Euclidian distance. The local fidelity is ensured by assigning to each new data point a weight that is the higher the closer it is to the original one. The artificial dataset is thus used to train an interpretable surrogate simple model like the linear regression instead of the original complex one. With reference to the linear model, the relevance of each feature on the initial individual prediction is thus defined by the coefficients found by solving the fitting. Together with SHAP, LIME is the most commonly used method to evaluate the impact of every single feature to a AI-derived result [42]. In this work, we used the Python implementation of LIME (https://github.com/marcotcr/lime) for tabular data. The surrogate model chosen was the linear regression. Despite the local nature of LIME, a global explanation for each index was provided by finding the LIME values for each subject of the validation set and thus calculating the average across all of them [43].

## III. Results

### A. Qualitative Assessment

**FIGURE 1.**
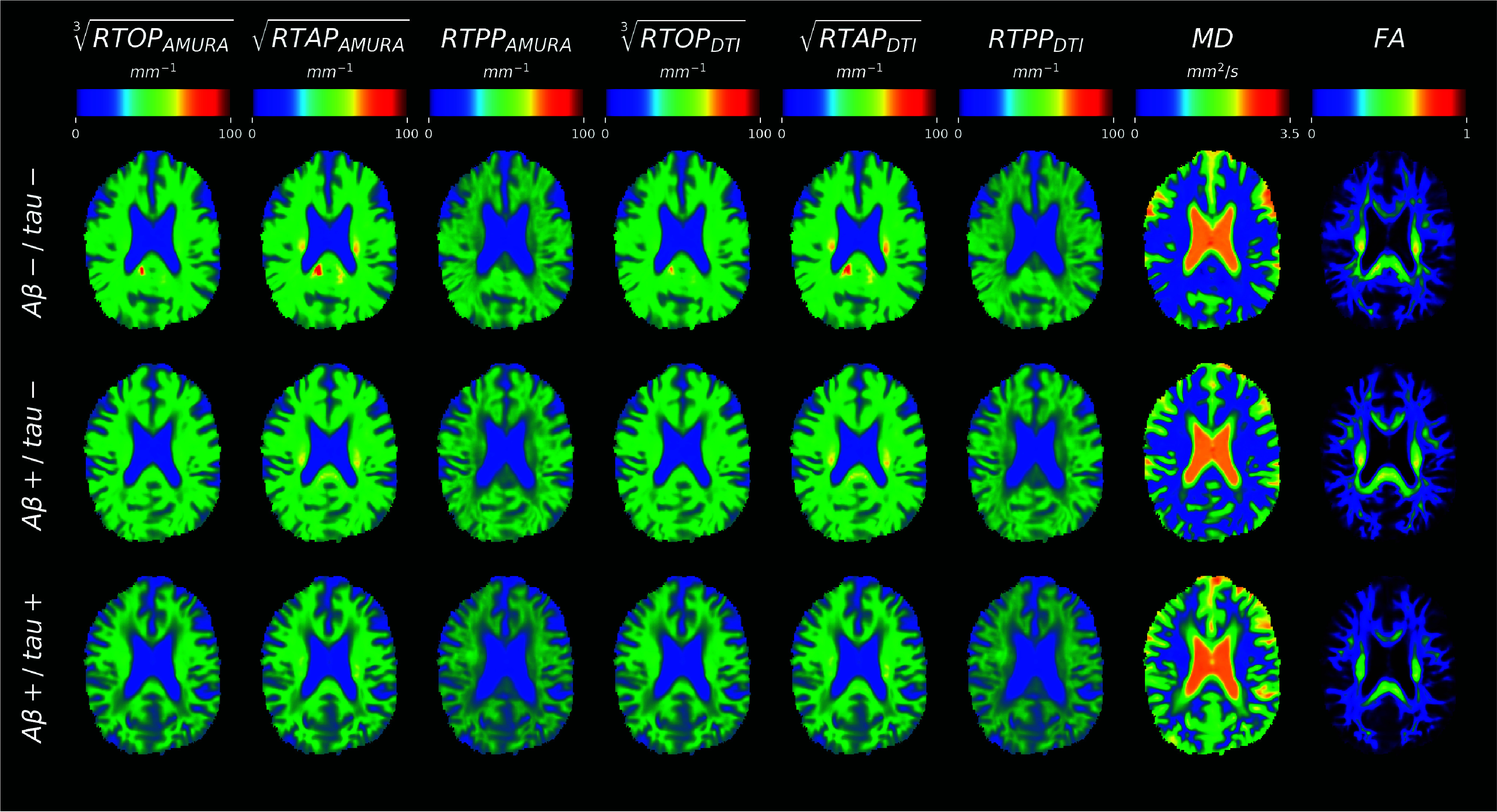
Microstructural maps of values of one representative subject for A $\beta -$/tau−, A $\beta +$/tau−, and A $\beta +$/tau+ groups (rows), respectively. The representative axial slices reported are in MNI space and are displayed in radiological convention. RTOP = return to the origin probability; RTAP = return to the axis probability; RTPP = return to the plane probability; MD = mean diffusivity; FA = fractional anisotropy.

The maps for each dMRI index for a representative subject of each group (*i.e.*, A $\beta -$/tau−, A $\beta +$/tau−, and A $\beta +$/tau+) are shown in Fig. 1. The cubic-root of RTOP, and the square-root of RTAP were calculated and reported to easily compare the three restriction indices (*i.e.*, RTOP, RTAP, and RTPP).

As expected, RTOP/RTAP/RTPP $_{\textrm {AMURA/DTI}}$ had similar contrast to FA, appearing hyperintense in regions where diffusion takes place preferentially along a single direction (*e.g., corpus callosum*). MD showed an opposite trend, reaching the highest values where diffusion is unrestricted. No evident differences across groups could be appreciated by qualitative assessment.

### B. TBSS Analysis

**FIGURE 2.**
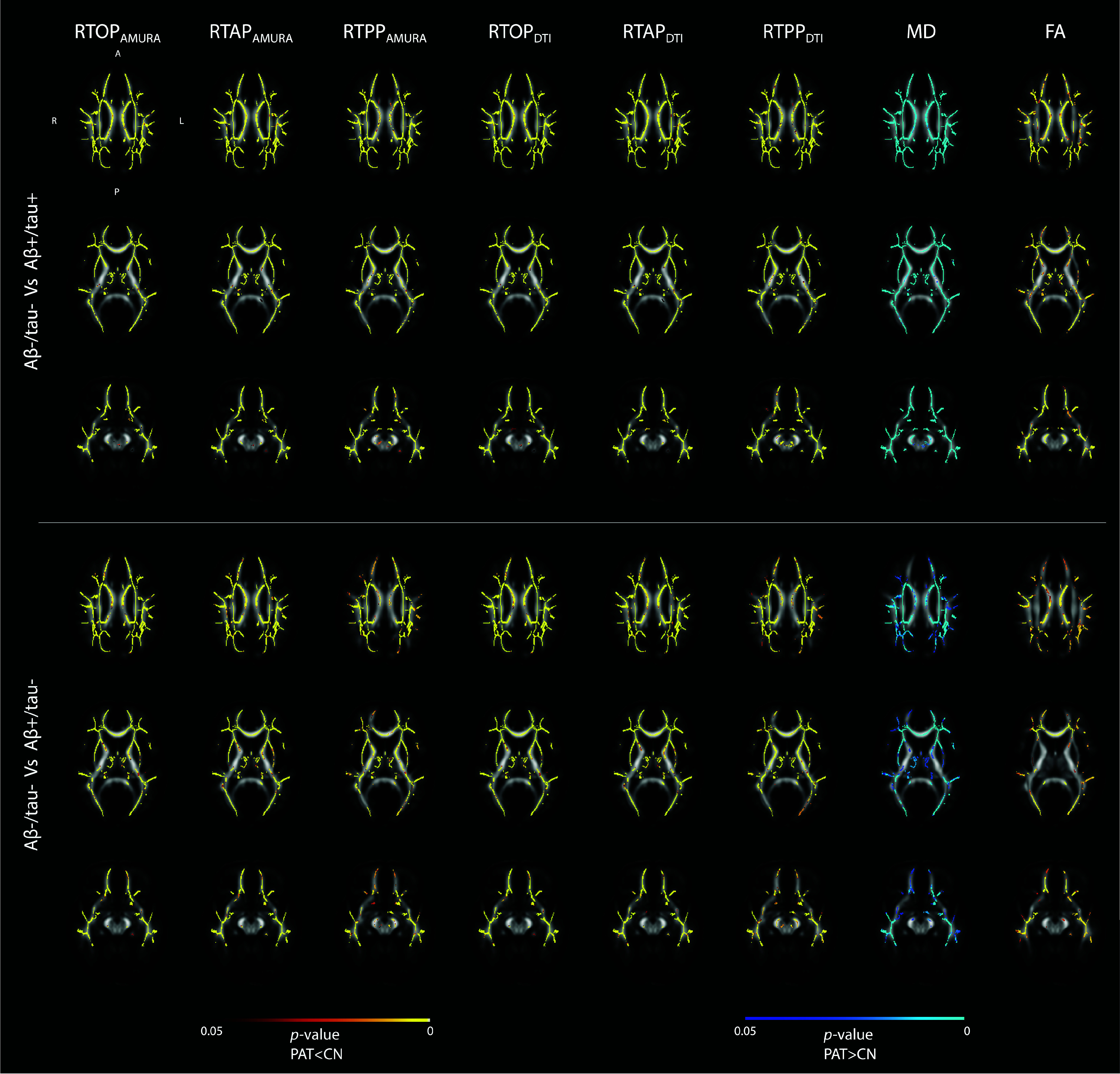
Tract-based Spatial Statistics (TBSS) results. The significant differences for both the contrasts A $\beta -$/tau− versus A $\beta +$/tau+ and A $\beta -$/tau− versus A $\beta +$/tau− are shown for the eight diffusion indices (columns). Significant voxels are superimposed on the FA image of the JHU DTI-based WM atlas. Red-yellow and blue-lightblue colormaps indicate statistically significant clusters corrected for multiple comparisons in the CN > patients and CN < patients contrasts, respectively (significant *p*-value =0.05).

The TBSS analysis results are shown in Fig. 2. The significant voxels for each of the considered indices are overlaid to the JHU-FA atlas. Results unveiled widespread statistically significant differences corrected for multiple comparisons (*p*-value $\leq 0.05$) between A $\beta $-/tau- and both A $\beta +$/tau− and A $\beta +$/tau+ groups for all indices. More in detail, RTOP/RTAP/RTPP $_{\textrm {AMURA/DTI}}$ and FA exhibited these significant differences only in the contrast A $\beta $-/tau- > A $\beta +$/tau− or A $\beta +$/tau+, while MD index displayed significance only in the opposite contrast.

### C. SVM Classification Performance

**FIGURE 3.**
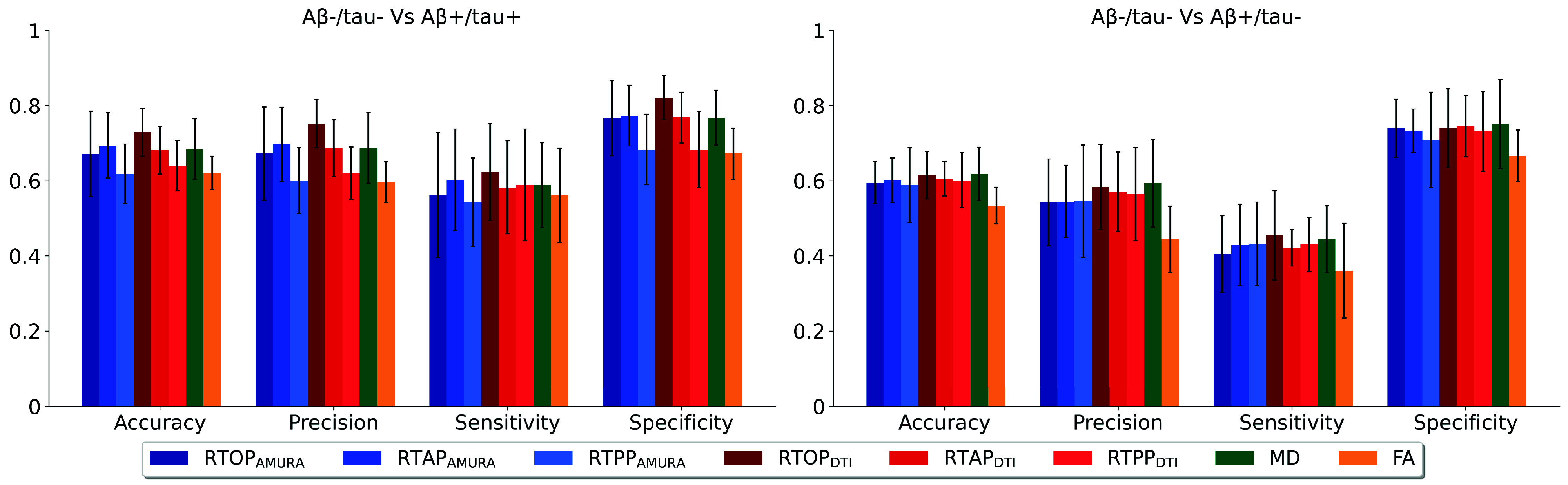
Mean accuracy, precision, sensitivity, and specificity, along with the relative standard deviation, across the 10 folds of the cross-validation step.

The SVMs performance is illustrated in Fig. 3, showcasing the mean and standard deviation of the measurements across the ten folds. The most challenging task involved discriminating A $\beta -$/tau− from A $\beta +$/tau− subjects. MD emerged as the best feature for distinguishing between these two classes, even though with a performance similar to that showed by AMURA and DTI. In particular, it resulted in an accuracy of 0.619. In this classification, the least effective performance was observed in the FA-based classification (accuracy =0.534). As anticipated, superior results were achieved in the A $\beta -$/tau− and A $\beta +$/tau+ condition. Specifically, $\textrm {RTOP}_{\textrm {DTI}}$ outperformed others, with an accuracy of 0.729, closely followed by $\textrm {RTAP}_{\textrm {AMURA}}$ with an accuracy of $0.694.~\textrm {RTPP}_{\textrm {AMURA}}$ demonstrated the poorest performance (accuracy =0.618). In both cases, the model was affected by a tendency toward imbalanced classification, sometimes labeling A $\beta +$/tau+ or A $\beta +$/tau- as A $\beta $-/tau-. This behavior is emphasized by the high specificity and relatively low sensitivity observed across the ten folds.

### D. XAI-Based Post-Hoc Assessment

**FIGURE 4.**
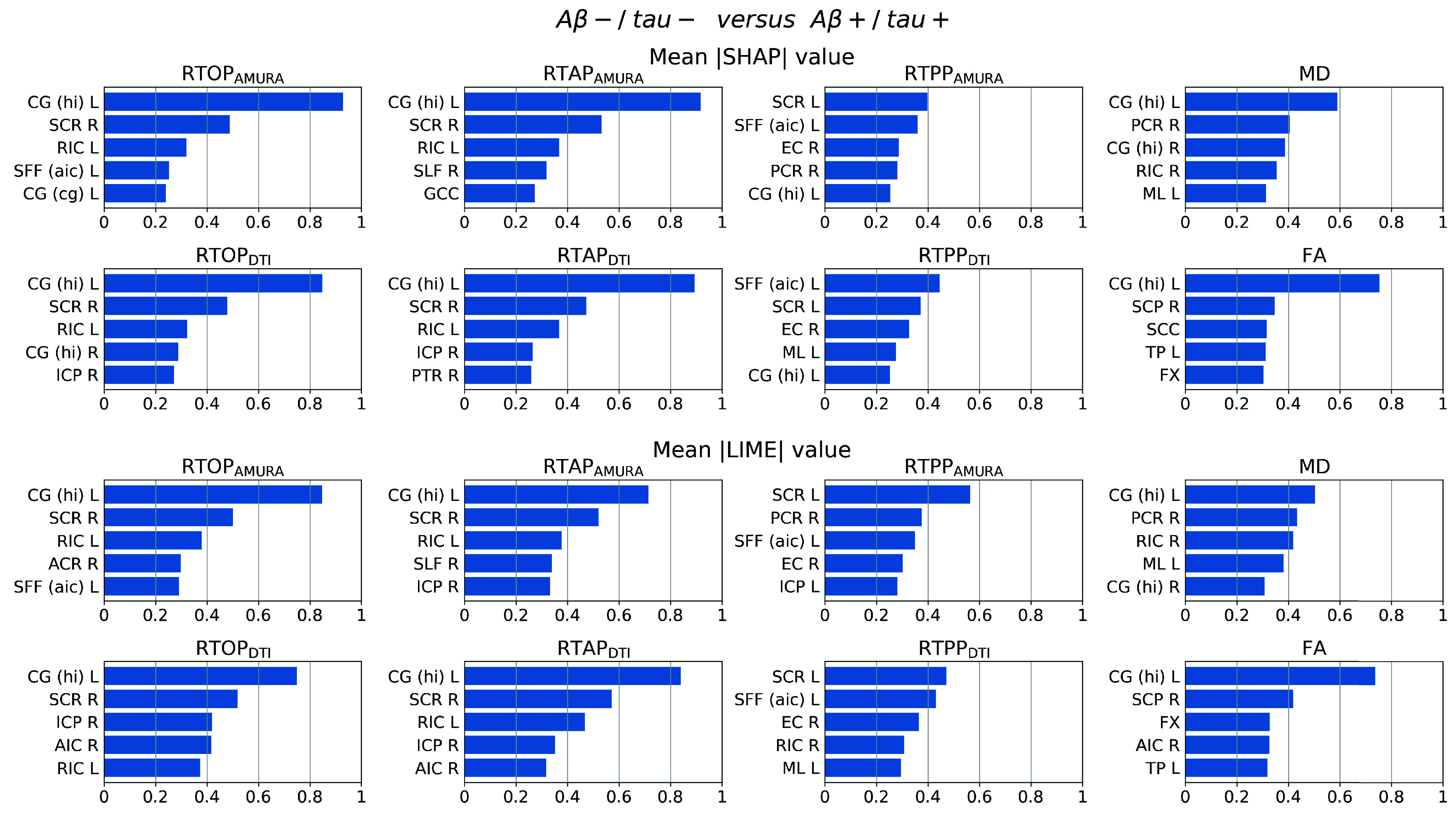
Mean SHAP (top) and LIME (bottom) value over the validation set for each feature and index-derived SVM classification; Abbreviations: left *cingulum* connecting the *cingulate gyrus* (CG (cg) L), left/right *cingulum* connecting the *hippocampus* (CG (hi) L/R), right *external capsule* (EC R), *column* and *body* of *fornix* (FX), *genu* of *corpus callosum* (GCC), right *inferior cerebellar peduncle* (ICP R), left *medial lemniscus* (ML L), right *posterior corona radiata* (PCR R), right *posterior thalamic radiation* including *optic radiation* (PTR R), left/right *retrolenticular part of internal capsule* (RIC L/R), *splenium* of *corpus callosum* (SCC), right *superior cerebellar peduncle* (SCP R), left/right *superior corona radiata* (SCR L/R), left *superior fronto-occipital fasciculus* that could be a part of *anterior internal capsule* (SFF (aic) L), right *superior longitudinal fasciculus* (SLF R), left *tapetum* (TP L), right *anterior corona radiata* (ACR R), right *anterior limb of internal capsule* (AIC R), left *inferior cerebellar peduncle* (ICP L).

Fig. 4 represents, for each dMRI index, the top five features found by SHAP and LIME mostly contributing to the classification task that reached the best performance (*i.e.*, A $\beta -$/tau− *versus* A $\beta +$/tau+). As evident, the findings from both the XAI methods are in agreement because at least four among the top five most impactful features are the same across the two approaches for each dMRI index. Only the sorting can vary slightly, and anyway it is preserved for the top two features except for RTPP. Of note, the left *cingulum* connecting *hippocampus* appeared as the most important WM tract to consider for distinguishing subjects with amyloid/tau positivity from negative ones. Indeed, in this WM tract, all microstructural indices except RTPP (in both AMURA and DTI versions) showed the highest SHAP value reflecting such a relevance. Instead, the discrepancy with RTPP was in agreement with its derived classification performance, which emerged as the worst. In addition, more generally, respectively RTOP, RTAP, and RTPP, demonstrated a high correspondence between the AMURA and DTI versions, often highlighting the same WM tracts with similar SHAP and LIME values.

## IV. Discussion

In this study, for the first time, we revealed the potential of RTOP/RTAP/RTPP $_{\textrm {AMURA}}$ as imaging markers for early AD detection by exploiting their WM characterization in subjects with amyloid and possibly tau pathology compared to subject without such a pathology. From a medical point of view, this could represent a further step toward a possible screening at a pre-clinical level without the need of more invasive methods. Pursuing this aim, we took advantage of both classical statistical (*i.e.*, TBSS) and machine learning techniques (*i.e.*, SVMs), using the well-established DTI-based indices as benchmark. Moreover, the SHAP and LIME XAI methods were employed to identify the features most contributing to the SVMs outcomes, enabling the translational value of the present work and identifying the *cingulum* WM tract as possible target for future clinical research trials. The usage of the two different XAI approaches served for comparing the outcomes and thus testing their reliability. However, the results were additionally critically analysed with respect to the literature to assess their plausibility.

Classical statistical analysis performed through TBSS further suggested the dMRI derived indices as possible imaging markers of microstructural degeneration from the earliest phases of AD. Indeed, widespread statistically significant differences between A $\beta $-/tau- and A $\beta $+/tau- or A $\beta $+/tau+ surviving the correction for multiple comparisons were found. In addition, the contrast of such significance evidenced a lower anisotropy and restriction along with a higher diffusivity in A $\beta $+/tau- or A $\beta $+/tau+ compared to A $\beta $-/tau-, compatible with a clinical picture of neurodegeneration and inline with literature [5].

In this study, alongside statistical analysis at the population level, we used AI to diagnose the pathology at the subject level. The A $\beta $/tau detection task is particularly complex because the mechanisms that lead to the development of AD are still unknown. Despite this, we anyway chose to use a relatively simple machine learning model, focusing instead on the type of information used for classification (*i.e.*, microstructural information). Indeed, the centrality of the role played by the goodness of the chosen features was made evident by the fact that even with a less complex model than the deep generative one used in [16] (*i.e.*, HexaGAN), our SVMs were able to achieve a similar performance to that obtained by Hwang et al. when based on T1w-MRI alone (*i.e.*, macrostructural information). Hwang and colleagues [16] were able to achieve a superior performance only at the cost of more input data than just the images.

SVM results interestingly revealed MD as the index leading to the highest accuracy when used to distinguish A $\beta -$/tau− *versus* A $\beta +$/tau− subjects, suggesting it as possible imaging marker of A $\beta $ irrespectively from the tau concentration.

Of note, a similar finding, although in gray matter (GM), was reported also by Spotorno et al. [4] employing the MAPMRI-derived mean squared displacement (MSD) index in GM. The MSD can be considered as the Ensemle Average Propagator (EAP)-based version of MD since both represent the average amount of diffusion in the unit time and, consequently, holds sensitivity to a lower or higher restriction [4], [44]. In [4], such a measure in GM was found to be correlated with many other markers of amyloid and tau pathology, but in particular the association with A $\beta $-PET and glial fibrillary acidic protein suggested its relationship to the astrocytic response to A $\beta $ aggregation.

However, in the present study, MD-related mean accuracy was lower compared to that of other indices in the classification of A $\beta -$/tau− versus A $\beta +$/tau+ subjects (accuracy = 0.685). More specifically, $\textrm {RTOP}_{\textrm {DTI}}$ and $\textrm {RTAP}_{\textrm {AMURA}}$ appear to be superior in performance, although not at the level of statistical significance, suggesting their sensitivity to the tau pathology onset (accuracy = 0.729 and 0.694, respectively). Also Chen et al. [3] observed that altered WM, as highlighted by their results using FA and MD, may reflect tau presence. In addition, they found a correlation with tau but not with A $\beta $ presence enforcing that finding. The present study provides additional evidence to such a hypothesis. All these findings witness in favor of designating indices like $\textrm {RTAP}_{\textrm {AMURA}}$ as possible better marker compared to the more standard FA and MD, inline with other results from Moody et al. [7], though these were obtained relying on MAPMRI on a multi-shell acquisition at higher *b*-values. Hence, according to our results, AMURA allows capturing fine microstructural modulations with a sensitivity comparable to MAPMRI but with data acquisitions requiring a lower number of samples.

In this study, XAI aided the decription of the SVM results, demonstrating one time more its important role when artificial intelligence is applied to medicine. Several works already showed the need of this tool, especially in pathology [45]. In the present work, the employment of XAI enabled the discovery of the *cingulum* WM tract as the most relevant to possibly detect early AD stage subjects. The validation of such a finding consists of its recovery as top feature through both SHAP and LIME, despite the substantial different principles at the basis of the two methods. In particular, by showing that different XAI methods led to the same interpretation of the results, we provided evidence of the stability of the explanations for each microstructural index. On the other hand, the importance of the features also depends on the ML model because of the peculiar assumptions at the basis of the model (e.g., linear relationships rather than others), algorithmic constraints (e.g., presence or absence of regularizations), etc. By studying the explanation that would be obtained by using a ML model different from SVM, it would be defined its so-called consistency [46]. Nevertheless, as also Molnar [46] observed, such an explanation property is controversial. Indeed, even though the algorithmic independence would reflect the robustness of the ranking and should be reached in ideal conditions, a direct comparison of the explanations across models should take into account the model’s complexity with respect to the numerosity of the samples, the impact of the different architectures, the sensitivity to noise and other factors affecting the performance in real conditions when the data is limited and noisy. For this reason, other architectures will be considered in future works while this work aimed at providing a framework ending in the explanation of the most immediate understanding for health and medicine screening applications.

In such a context, we further confirmed the impact of the *cingulum* by looking for studies in literature which emphasized its role. Microstructural alterations in this tract were found in MCI and individuals at genetic risk or family predisposition for AD [5]. It was also found significantly altered when specifically investigated in subjects with pathological levels of tau presence compared to CN [47]. Very recently, also Chen et al. [3] reported the central role of the *cingulum* tract when investigating tau pathology. More in detail, in addition to microstructural alterations, they found a significant correlation of these changes with tau burden in the AD continuum. Interestingly, the *cingulum* is one of the WM tracts most implicated in episodic memory function, that is known to be tipycally impaired in AD. This can be considered as an added form of validation of SHAP and LIME outcomes through literature-based plausibility assessment.

Future works will include other objective assessment methods such as association studies and analyses of the impact of features collinearity. The former are intended to characterize the biological differences in terms of microstructure as depicted by FA, MD, etc. and other terms like functional connectivity; emerging studies in this direction are [32] and [34]. The latter, instead, are aimed at addressing the possible bias on the models’ performance due to the collinearity potentially present in datasets with a high number of features; for example, there exist methods specifically tailored for SHAP [48], [49] or proxies like the modified informative position and the normalized movement ratio formalized by Salih et al. [50], [51] that can be used to quantify the robustness of the feature importance provided by XAI methods with sensitivity with respect to the presence of collinearity.

Concerning the investigation of AMURA model’s potential, the trend’s proximity of its derived indices to their DTI counterparts confirmed the robustness of their characterization also in amyloid/tau pathological tissue. However, additional investigations using data acquired with higher *b*-value but still clinically feasible number of samples would be required to fully exploit this model. The expectation is to derive indices better approximating those based on the EAP (*e.g.*, MAPMRI-like) with well-known greater sensitivity compared to the ones based on DTI [11]. Moreover, following [17], a future work could include other AMURA indices like the moment-based representations of the diffusion process in brain tissues.

## V. Conclusion

This study investigated for the first time AMURA in the characterization of amyloid and tau pathology in AD, revealing their potential as imaging markers for a timely diagnosis relying on SVM classification and XAI-based interpretation of the outcomes. In a translational perspective, findings highly suggest for future clinical works focusing on *cingulum* WM tract analysed through non-invasive dMRI data acquired with high *b*-value but still reduced protocol as enabled by AMURA.
